# Epimedin A inhibits the PI3K/AKT/NF-κB signalling axis and osteoclast differentiation by negatively regulating TRAF6 expression

**DOI:** 10.1186/s10020-024-00893-w

**Published:** 2024-08-16

**Authors:** Jun Li, Jia J. Wei, Cen H. Wu, Tao Zou, Hong Zhao, Tian Q. Huo, Cheng J. Wei, Ting Yang

**Affiliations:** 1 Department of Spine Surgery, Changzhou TCM Hospital Affiliated to Nanjing University of Chinese Medicine, Changzhou, 213000 People’s Republic of China; 2 Department of Orthopedics, Yunnan Province Hospital of Traditional Chinese Medicine, Kunming, 650000 People’s Republic of China; 3 Department of Orthopedics, Jiangsu Province Hospital of Traditional Chinese Medicine Affiliated to Nanjing Universityof Chinese Medicine, Nanjing, 210000 People’s Republic of China; 4 Department of Rheumatology, Changzhou TCM Hospital Affiliated to Nanjing University of Chinese Medicine, Changzhou, 213000 People’s Republic of China

**Keywords:** Epimedin A, Osteoporosis, Osteoclastogenesis, TRAF6, PI3K/AKT/NF-κB signalling pathway

## Abstract

**Background:**

Epimedin A (EA) has been shown to suppress extensive osteoclastogenesis and bone resorption, but the effects of EA remain incompletely understood. The aim of our study was to investigate the effects of EA on osteoclastogenesis and bone resorption to explore the corresponding signalling pathways.

**Methods:**

Rats were randomly assigned to the sham operation or ovariectomy group, and alendronate was used for the positive control group. The therapeutic effect of EA on osteoporosis was systematically analysed by measuring bone mineral density and bone biomechanical properties. In vitro, RAW264.7 cells were treated with receptor activator of nuclear factor kappa-B ligand (RANKL) and macrophage colony-stimulating factor (M-CSF) to induce osteoclast differentiation. Cell viability assays, tartrate-resistant acid phosphatase (TRAP) staining, and immunofluorescence were used to elucidate the effects of EA on osteoclastogenesis. In addition, the expression of bone differentiation-related proteins or genes was evaluated using Western blot analysis or quantitative polymerase chain reaction (PCR), respectively.

**Results:**

After 3 months of oral EA intervention, ovariectomized rats exhibited increased bone density, relative bone volume, trabecular thickness, and trabecular number, as well as reduced trabecular separation. EA dose-dependently normalized bone density and trabecular microarchitecture in the ovariectomized rats. Additionally, EA inhibited the expression of TRAP and NFATc1 in the ovariectomized rats. Moreover, the in vitro results indicated that EA inhibits osteoclast differentiation by suppressing the TRAF6/PI3K/AKT/NF-κB pathway. Further studies revealed that the effect on osteoclast differentiation, which was originally inhibited by EA, was reversed when the TRAF6 gene was overexpressed.

**Conclusions:**

The findings indicated that EA can negatively regulate osteoclastogenesis by inhibiting the TRAF6/PI3K/AKT/NF-κB axis and that ameliorating ovariectomy-induced osteoporosis in rats with EA may be a promising potential therapeutic strategy for the treatment of osteoporosis.

## Background

Osteoporosis is the most prevalent metabolic bone disorder and is predominantly characterized by skeletal fractures, which result in substantial risks of morbidity and mortality as well as a major socioeconomic burden on society (Efthimiou 2020). Dysregulation of the intricate balance between osteoclasts and osteoblasts, which leads to disruptions in bone homeostasis, can precipitate osteolytic conditions such as osteoporosis (Leali et al. 2009). Therefore, osteoclasts play a pivotal role in bone resorption and subsequent bone loss, making them prime targets for therapeutic intervention in osteoporosis. The elucidation of osteoclast signalling pathways thus offers a promising avenue for the development of novel drugs aimed at impeding osteoclast differentiation and function (Cheng et al. 2021).

Research has highlighted the indispensable role of receptor activator of nuclear factor-κB ligand (RANKL) and macrophage-colony stimulating factor (M-CSF), which are secreted by osteoblasts, in osteoclast differentiation (Dou et al. 2021). After RANKL binds to its receptor RANK, downstream signalling pathways, such as the NF-κB pathway, are activated, leading to the expression of osteoclast-related genes, including nuclear factor of activated T cells (NFATc1), c-fos, ATPase H+ transporting V0 subunit d2 (ATP6V0d2), and dendritic cell-specific transmembrane protein (DC-STAMP) (Dou et al. 2021; Yun et al. 2019). Blocking the RANK/RANKL pathway can inhibit osteoclast differentiation, promote bone remodelling, prevent bone resorption, maintain bone mineral density, and reduce the risk of fracture in myeloma and other diseases (Yee and Raje 2012). Downregulation of TRAF6 has similarly been shown to curb bone resorption and osteoclast activation in murine osteoporosis models (Zeng et al. 2020).

**Fig. 1:**
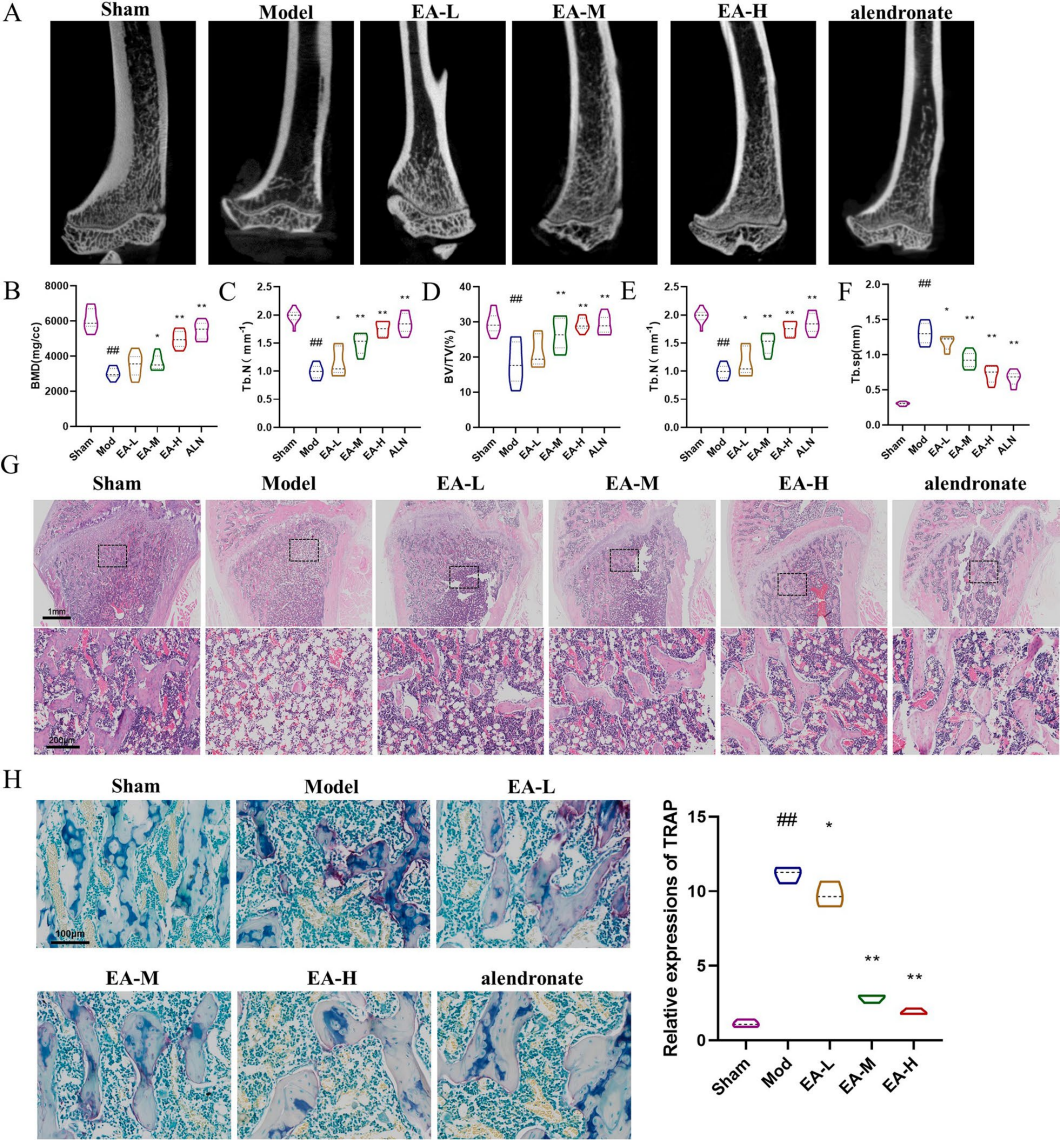
EA attenuates ovariectomy-induced bone loss in vivo. **a** Microscopic CT analysis of the distal femur in each group: sham group, model group, EA-L, EA-M and EA-H groups, and ALN (alendronate, positive drug) group. n = 12 for experimental replicates in each group. **b**–**f** Quantitative analysis of BMD, Tb. Th, BV/TV, Tb.N, and Tb.Sp. n = 12 for experimental replicates in each group. **g** Representative images of HE staining of distal femur sections. Scale bar = 100 and 200 µm. n = 12 for experimental replicates in each group. **h** Representative image of a femur stained with TRAP. Scale bar = 100 μm. n = 12 for experimental replicates in each group. ^##^P < 0.01 vs. the sham group, ^#^P < 0.05 vs. the sham group; **P < 0.01 vs. the model group, *P < 0.05 vs. the model group

Epimedin A (EA, Fig. 1a), a prenylated flavanol glycoside, is one of the primary active constituents in Herba Epimedii, traditional Chinese herbs long utilized for strengthening bones, muscles, and tendons and to treat rheumatism, cardiovascular disease, diabetes, infertility, and amnesia (Liu et al. 2021; Zhang et al. 2008). Recent evidence has increasingly highlighted EA’s potential to mitigate osteoclast differentiation and bone resorption, although its underlying mechanism remains unclear (Tosteson et al. 2003; Wang et al. 2016). Consequently, we studied the effect of EA on osteoclast differentiation and its potential mechanism in cells and rats.

## Methods

### Materials and reagents

Epimedin A (EA, Cas: 110623-72-8, IE0160, purity ≥ 97.0%) and alendronate sodium (ALN, Cas: 121268-17-5, A6780, purity ≥ 97.0%) were purchased from Solarbio (Beijing, China). Antibodies specific for NFATc1 (ab2796), receptor activator of nuclear factor κB ligand (RANKL, ab239607), RANK (ab305233), NF-κB p65 (ab32536) and p-NF-κB p65 (phospho S536, ab76302) were purchased from Abcam (Cambridge, MA, USA). Other antibodies, including anti-TRAF6 (#67591), anti-MAPK P-P38 (Thr180/Tyr182, #4511), anti-MAPK p38 (#8690), P-AKT (Ser473, #4060), anti-AKT (#4685), anti-GAPDH (#5174), and anti-rabbit IgG (H+L) (#14708), were purchased from Cell Signaling Technology (Danvers, MA, USA). All antibodies were used at the recommended concentrations in accordance with their protocols, as described previously.

### Animals

A total of 60 female Wistar rats (240 ± 20 g, 10–12 weeks old) were provided by SPF (Beijing) Biotechnology Co., Ltd. (Beijing, China). All rats were house in a temperature-controlled environment with a 12-h light–dark cycle. All animal experimental procedures complied with the guidelines for the Animal Care and Experiment Committee of Changzhou Hospital of Traditional Chinese Medicine.

### Osteoporosis rat model and treatment

Seventy-two rats underwent a 1-week adaptation period after which a proportion of the rats (n = 12) underwent sham surgery, while the remaining rats (n = 60) underwent surgical removal of the ovaries. After anaesthesia via intraperitoneal injection of 3% sodium barbiturate (40 mg/kg), the surgical site was shaved, and a lateral incision was made from the mid-axillary line of the quarter rib area to the spinal area. Bilateral ovaries were then excised entirely to induce the osteoporosis model. The sham-operated group underwent the same procedure but without bilateral ovary removal. Subsequently, wound closure was performed for each group of rats, followed by the application of erythromycin ointment to prevent infection. Vaginal smear examinations commenced from the 2nd postoperative day, with continuous daily observations over a week. Rats displaying consistent oestrous cycle changes were considered to have undergone successful ovarian removal. The ovariectomized (OVX) rats were randomly allocated into five groups: (1) model group; (2) EA-L group (5 mg/kg/d epimedin A); (3) EA-M group (10 mg/kg/d epimedin A); (4) EA-H group (20 mg/kg/d epimedin A); and (5) ALN group (2.5 mg/kg/d alendronate). Epimedin A and ALN were dissolved in normal saline and orally administered to the rats daily for 90 days.

### Bone loss assay

At the end of experiments, all rats underwent euthanasia. The left thighbone was subjected to high-resolution micro-CT (Skyscan 1176, Aartselaar, Belgium), which was used to generate a three-dimensional finite element model of the rat thighbone with MIMICS software. For each sample, standard bone parameters, including bone mineral density (BMD), bone volume/tissue volume ratio (BV/TV), trabecular thickness (Tb.Th), trabecular number (Tb.N), and trabecular separation (Tb.Sp), were examined and analysed as previously described (Kular et al. 2012).

### Morphometric analysis of bone tissues

The right thighbone of the rats was fixed in 4% paraformaldehyde, decalcified by 10% EDTA, paraffin-embedded and cut into 5 μm sections. Then, these sections were used for morphometric analysis of bone tissues with haematoxylin and eosin and TRAP staining. The quantitative parameters, such as osteoclast surface per bone surface (OcS/BS), the ratio of bone volume/tissue volume (BV/TV), and number of osteoclasts per bone perimeter (N.Oc/BS), were analysed using Bioquant Osteo software (Nashville, USA).

### Construction of the osteoclast model

RAW264.7 cells purchased from the American Type Culture Collection (Rockville, MD) were cultured in DMEM supplemented with 10% foetal bovine serum, 2 mM l-glutamine and 100 U/mL penicillin/streptomycin. RAW264.7 cells were cultured in 50 ng/mL RANKL and 10 ng/mL M-CSF, the medium was changed every 3 days, and RANKL was added to induce osteoclast differentiation. Six days after induction, TRAP staining was performed to observe the morphology of multinucleated osteoclasts after induction.

### Cell viability assay

The effect of EA on osteoclastogenesis was determined using a CCK-8 assay kit (Dojindo Molecular Technology, Kumamoto, Japan). RANKL-induced RAW264.7 cells were inoculated at a density of 2.5 × 10^3^ cells/well and cocultured with EA at different concentrations (0.1 µmol/L, 0.2 µmol/L and 0.4 µmol/L). Two days later, the RAW264.7 cells were incubated in fresh medium containing 10 μL of CCK-8 solution for 2 h, and the optical density (OD) was measured at a wavelength of 450 nm using a microplate with an enzyme.

### In vitro experimental grouping

RAW264.7 cells pretreated with RANKL/M-CSF were divided into an EA-treated group and an EA-free group (control group). RAW264.7 cells cultured with RANKL/M-CSF were treated with 0.1 μM EA-L, 0.2 μM EA-M, or 0.4 μM EA-H for 5 days at 37 °C. Then, the cells in each group were subjected to a cell viability assay and subsequent experiments.

### Construction and infection of lentivirus

A TRAF6 interference sequence (TRAF6-siRNA, 22,034) was synthesized by General Biol (Anhui) Co., Ltd. (Hefei, China), and inserted into the hU6-MCS-CMV-puromycin vector. Then, the plasmids were transfected into 293T cells. RAW264.7 cells were infected with lentivirus-mediated TRAF6-siRNA or its negative control lentivirus (control-siRNA) for 48 h, and then, the cells were changed to complete optimal medium without lentivirus. At 72 h after infection, the RNA interference efficacy of RAW264.7 cells was determined by Western blot analysis. After RAW264.7 cells were infected with lentivirus (TRAF6-siRNA or control-siRNA), they were also treated with RANKL/M-CSF to induce differentiation in the absence or presence of epimedin A.

### TRAP staining

RAW264.7 cells were cultured in 96-well plates with varying concentrations of epimedin A in the presence of RANKL/M-CSF. After differentiation into osteoclasts, the cells were fixed in 4% paraformaldehyde for 10 min, washed with cold PBS, and stained with a tartrate-resistant acid phosphatase kit (P0332, Beyotime) following the manufacturer’s instructions. Cells positive for TRAP, which indicated mature osteoclasts, were counted under a microscope.

### Immunofluorescence assay

The RAW264.7 cells of each group were grown on glass coverslips to measure the relative expression of the intracellular protein NFATc1. In summary, 4.0% paraformaldehyde in PBS was used to fix the osteoclasts for 30 min. After three PBS washes, the cells were permeabilized for 5 min with 0.25% Triton X-100 (v/v), and then, they were blocked for 1 h in PBS containing 3% bovine serum albumin. TRITC rhodamine-conjugated phalloidin was used to stain the F-actin rings for 30 min, while DAPI solution was used to stain the nuclei for 30 s at room temperature. Finally, after RAW264.7 cells were washed with PBS, fluorescence images were captured and viewed using an Olympus BX51 fluorescence microscope.

### Quantitative real-time PCR

The grown cells of each group underwent three rounds of washing in cold PBS before being lysed with TRIzol reagent (Invitrogen, Thermo Fisher Scientific, Inc.). After isolation of total RNA in accordance with the manufacturer's instructions, cDNA was produced by reverse transcription. Next, real-time PCR was used to quantify the differential expression of specific genes (normalized by the GAPDH level); this process was performed using an ABI-7500 system (Applied Biosystems, CA, USA). The reaction mixture consisted of 2 μL of cDNA, 20 μL of SYBR Mixture (TaKaRa, Liaoning, China), 16 μL of ddH2O, 1 μL of forward primer (10 μM), and 1 μL of reverse primer (10 μM). The cycling conditions were as follows: 95 °C for 10 min, 35 cycles of 15 s at 95 °C and 1 min at 60 °C for 1 min, and a final step at 4 °C for 10 min. NFATc1: F (5ʹ-3ʹ)-AACTTTCTGCAAGACTCCAA, R (5ʹ-3ʹ)-TTATTCTCTGGTTGCGGAAA; CTSK: F (5ʹ-3ʹ)-ATGAAATCTCTCGGCGTTTA, R (5ʹ-3ʹ)-GAGAGGCCTCAAGATTATGG; OSCAR: F (5ʹ-3ʹ)-CTCCAGCTGTCTACTCTCTGTG, R (5ʹ-3ʹ)-TAGGGGCACTGGTGATGTG; GAPDH: F (5ʹ-3ʹ)-GGTCCCAGCTTAGGTTCAT, R (5ʹ-3ʹ)-CCTTCACCATTTTGTCTACG.

### Western blot

The cell cultures were supplemented with 200 μL of RIPA lysis buffer containing 2 μL of PMSF (Thermo, USA). After centrifugation and ultrasonography of the cell solution on ice, the supernatant was obtained, and a BCA assay kit (Solarbio, Beijing, China) was used to measure the protein content. The protein samples were then electrophoresed one after the other for 2 h at 80 V. The proteins were then transferred to nitrocellulose membranes for 1.5 h at a constant flow rate of 200 mA. Primary and matching secondary antibodies were used for Western blotting. The relative protein levels were standardized to that of tubulin as an internal control. ImageJ software was used to quantify the density of the protein bands.

### Statistical analysis

Each set of data is presented as the mean ± standard deviation (SD) and is representative of at least three separate studies. Normally distributed data between groups (Brown-Forsythe test) were analysed using one-way analysis of variance (ANOVA), and Tukey’s post hoc test was used if there were overall differences.

## Results

### EA alleviates ovariectomy-induced bone loss in vivo

To investigate the potential protective effects of epimedin A (EA) against osteoporosis, we utilized a ovariectomized rat model to simulate postmenopausal osteoporosis. The model group exhibited substantial bone loss, ALN significantly alleviated osteoporosis in rats, and the administration of EA by gavage also substantially attenuated ovariectomy-induced distal femoral bone loss (Fig. 1a). As shown in Fig. 1b–f, significant increases in the BMD, Tb.Th, BV/TV, Tb.N and Tb.Sp in the EA groups were observed compared to those in the model group. Histological analysis via H&E staining (Fig. 1g) corroborated these findings, revealing diminished cortical thickness and bone volume, as well as compromised trabecular microstructure, in the model group compared to the control group. Compared with the model group, the EA group exhibited high and intact trabecular density, a regular arrangement, and improved trabecular fracture and thinning. To further explore whether the osteoprotective effect of EA was achieved by decreasing the osteoclast reduction and activity, we analysed TRAP expression in the femur by immunofluorescence. TRAP expression was upregulated in OVX rats, and the upregulation of TRAP expression was significantly reduced by EA stimulation (Fig. 1h). In conclusion, these results suggest that EA is effective in rescuing ovariectomy-induced bone loss and increases in osteoclasts.

### EA inhibits RANKL-induced osteoclast differentiation in vivo

**Fig. 2:**
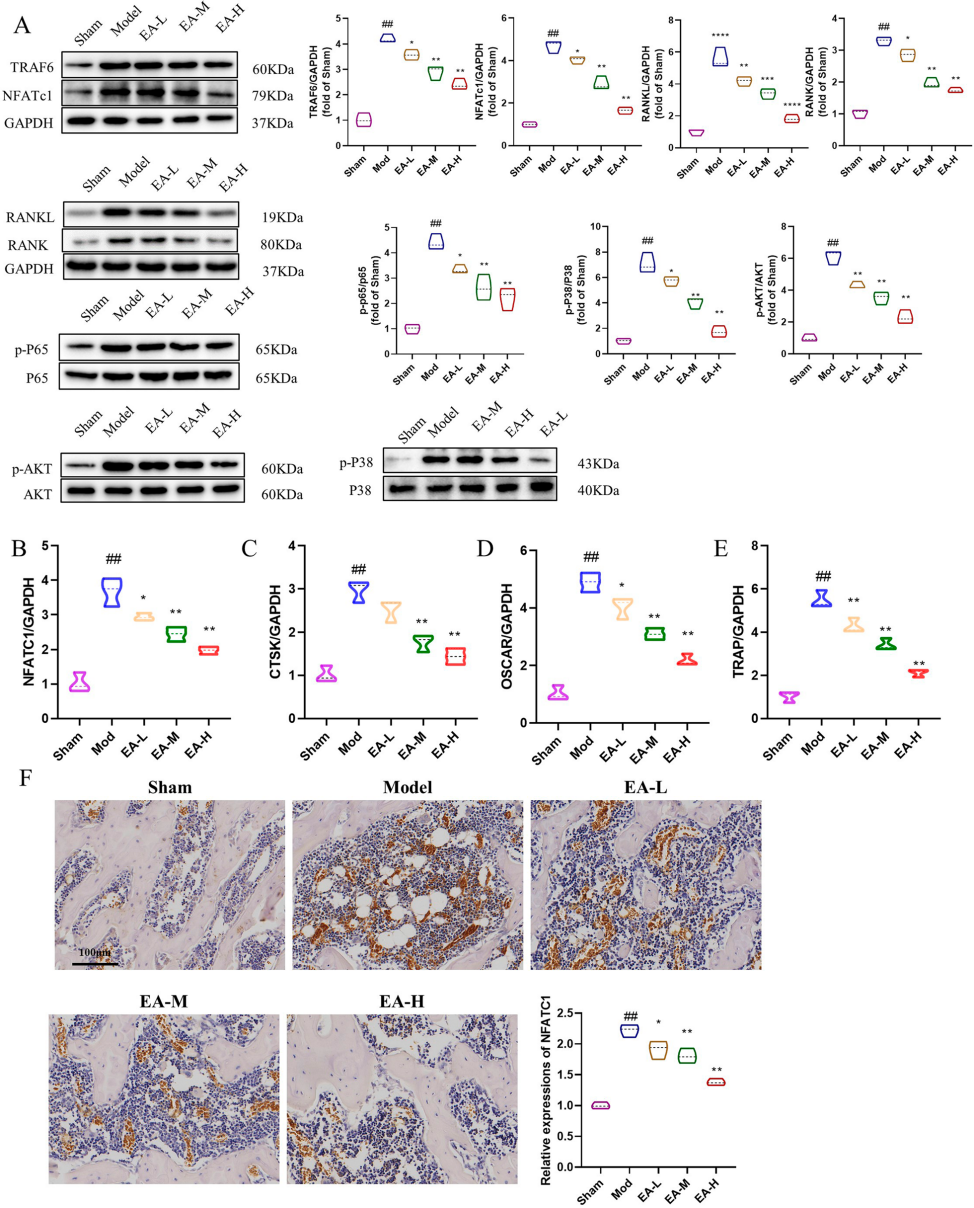
EA inhibits osteogenic differentiation of OCs through the NF-κB/MAPK signalling pathway in vivo. **a** WB evaluation of the protein expression of TRAF6, NFATc1, RANKL, RANK, NF-κB p-P65/P65, p-AKT/AKT and MAPK p-P38/P38. n = 3 for experimental replicates in each group. **b**–**e** The mRNA expression of the osteoclast-related genes NFATc1, Ctsk, Oscar, and Trap was determined by qPCR. n = 3 for experimental replicates in each group. **f** Immunohistochemical analysis of NFATc1. Scale bar = 50 μm. n = 3 for experimental replicates in each group. ^##^P < 0.01 vs. the sham group, ^#^P < 0.05 vs. the sham group; **P < 0.01 vs. the model group, *P < 0.05 vs. the model group

Research findings indicate that the Akt/NF-κB pathway is pivotal for regulating osteoblast differentiation and viability. Consequently, we aimed to determine whether EA hinders osteoblast differentiation via modulation of the Akt/NF-κB signalling cascade. As shown in Fig. 4a, Western blot analysis revealed that EA attenuated Akt and NF-κB phosphorylation, underscoring its significant inhibitory effect on the Akt/NF-κB signalling pathway. Moreover, the expression levels of TRAF6 and NFATc1 were reduced. The mRNA expression of the tibial osteoclast-related genes NFATc1, Ctsk, Oscar, and Trap was also reduced by EA (Fig. 2b–e). The immunohistochemical analysis of NFATc1 also provided further evidence of suppressed osteoclastogenesis (Fig. 2f).

### EA inhibits RANKL-induced osteoclastogenesis in vitro

**Fig. 3:**
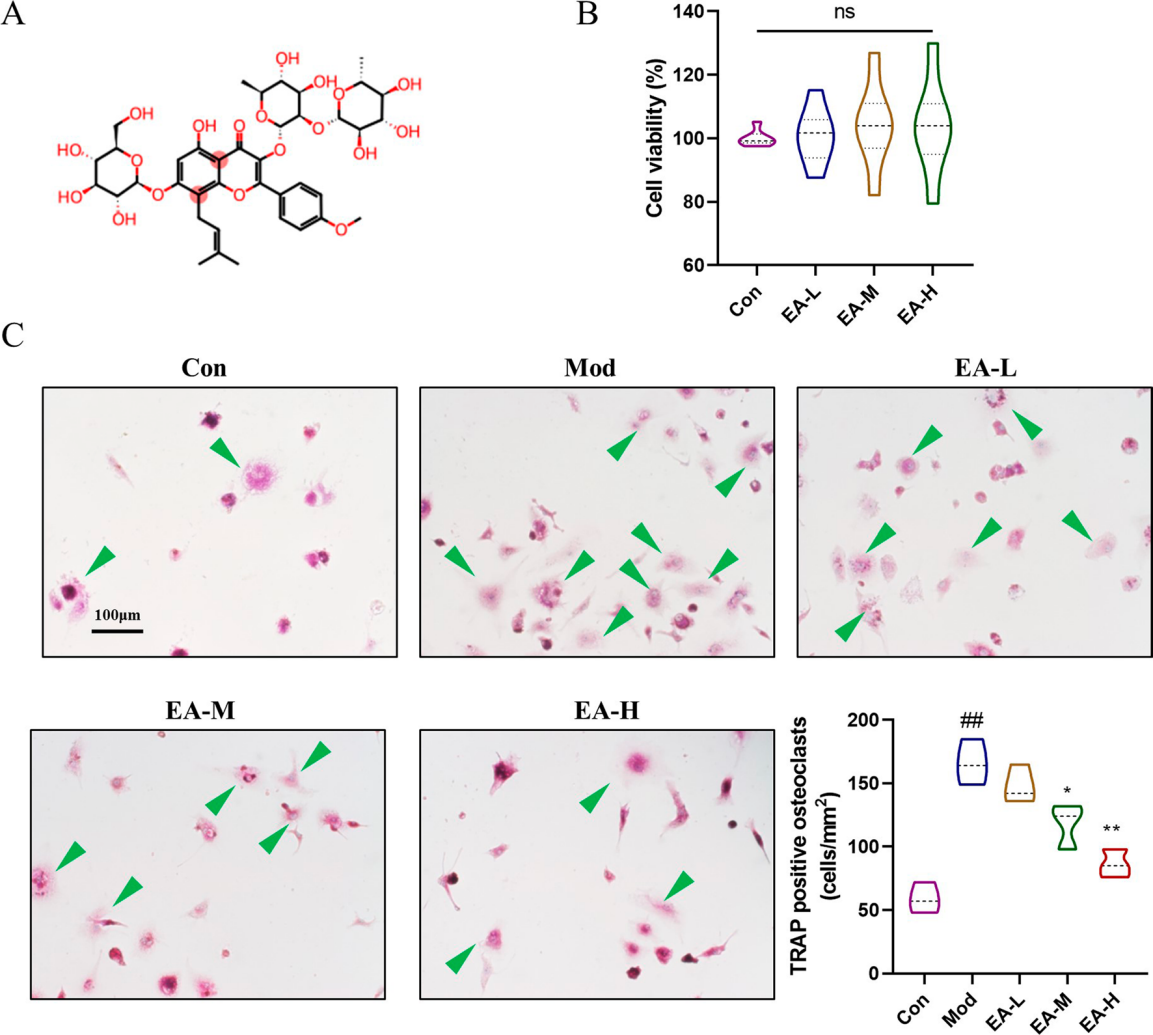
High concentrations of EA decrease OC viability and differentiation. **a** Chemical structure and molecular formula of EA. **b** Effect of EA on the viability of OCs as determined by CCK-8 assays. n = 6 for experimental replicates for each group. **c** Osteoclast differentiation was detected by Trap staining. n = 3 for experimental replicates for each group. ^##^P < 0.01 vs. the control group, ^#^P < 0.05 vs. control group; **P < 0.01 vs. the model group, *P < 0.05 vs. the model group

To investigate the potential cytotoxic effects of EA on osteoclasts, we conducted cell viability assays using RAW 264.7 cells, which are derived from murine macrophages, to determine the optimal drug dosage. As shown in Fig. 3b, EA did not impede cell proliferation, even at concentrations as high as 0.1 μM, 0.2 μM, and 0.4 μM (p > 0.05). Subsequently, to further examine the impact of EA on osteoclast formation, we coincubated RAW264.7 cells with selected concentrations of EA and RANKL/M-CSF throughout osteoclastogenesis. As shown in Fig. 1c, the RANKL group exhibited a significant increase in the formation of TRAP-positive multinucleated osteoclasts compared to the control group, whereas EA treatment effectively inhibited RANKL-induced osteoclastogenesis, as shown by the reduction in the number of osteoclasts. These findings collectively suggest that EA suppresses osteoclastogenesis in a concentration-dependent manner without inducing apparent cytotoxic effects.

### EA decreases RANKL-induced osteoclast-specific protein expression in vitro

**Fig. 4:**
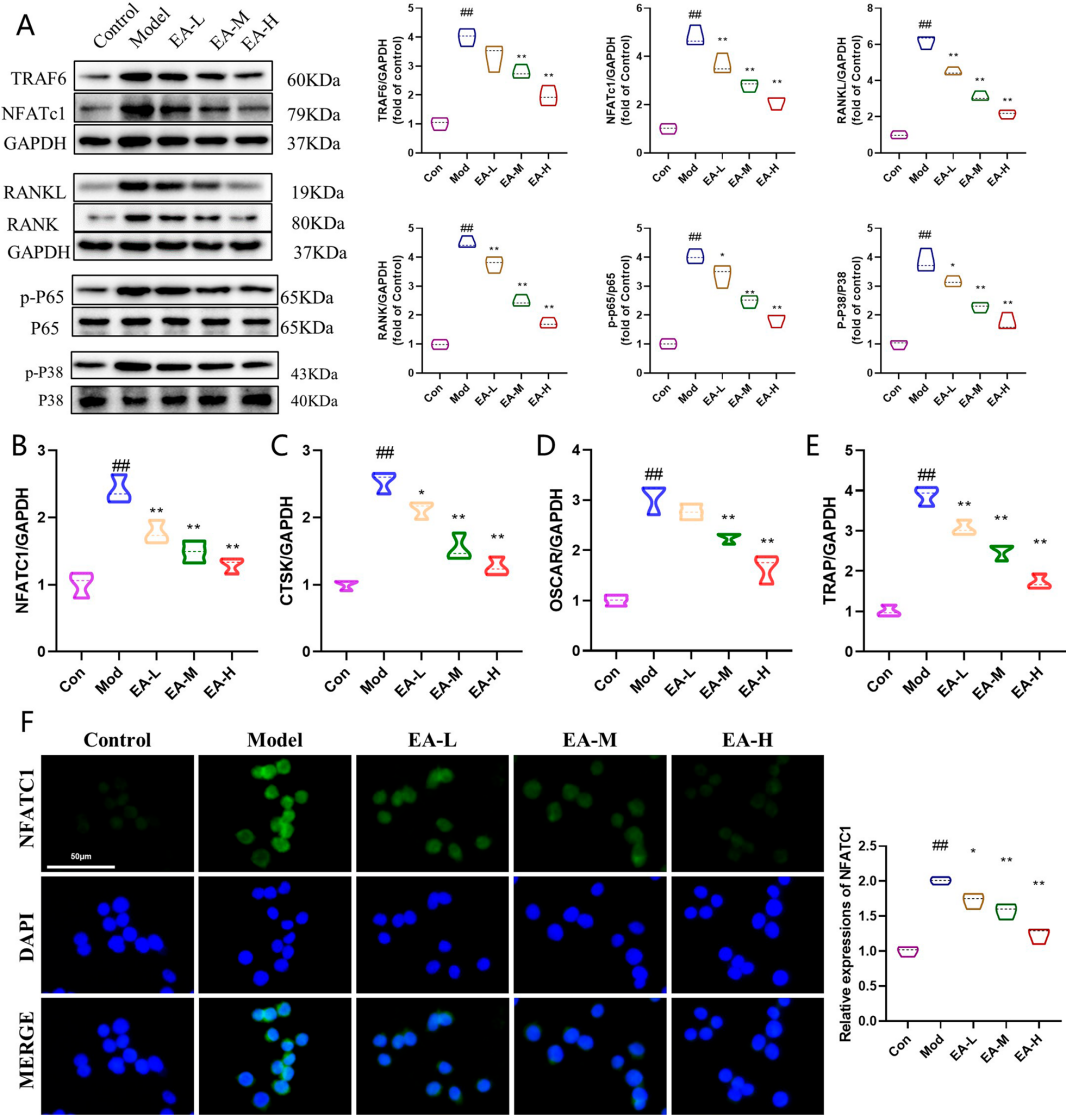
EA inhibits osteogenic differentiation of OCs through the NF-κB/MAPK signalling pathway in vitro. **a** WB evaluation of the protein expression of TRAF6, NFATc1, NF-κB p-P65/P65, and MAPK p-P38/P38. n = 3 for experimental replicates in each group. **b**–**e** The mRNA expression of the osteoclast-related genes NFATc1, Ctsk, Oscar, and Trap was determined by qPCR. n = 3 for experimental replicates in each group. **f** Immunofluorescence analysis of NFATc1. Scale bar = 50 μm. n = 3 for experimental replicates in each group. ^##^P < 0.01 vs. the control group, ^#^P < 0.05 vs. the control group; **P < 0.01 vs. the model group, *P < 0.05 vs. the model group

To further investigate the positive effects of EA on the formation of osteoclasts, we assessed the protein expression levels of TRAF6 and osteoclast-related genes, including NFATc1, phosphorylated NF-κB/NF-κB, and phosphorylated MAPK/MAPK. As shown in Fig. 4a, the Western blotting results demonstrated that EA treatment led to a concentration-dependent decrease in the expression levels of these osteoclast-related proteins. The Western blot analysis findings were consistent with those obtained from the quantitative PCR analysis, as shown in Fig. 4b–e. As shown in Fig. 4f, the immunofluorescence results confirmed that EA inhibited NFATc1-mediated osteoclast formation.

### EA regulates the Nrf2/NF-κB pathway to inhibit osteoclastogenesis in vitro

**Fig. 5:**
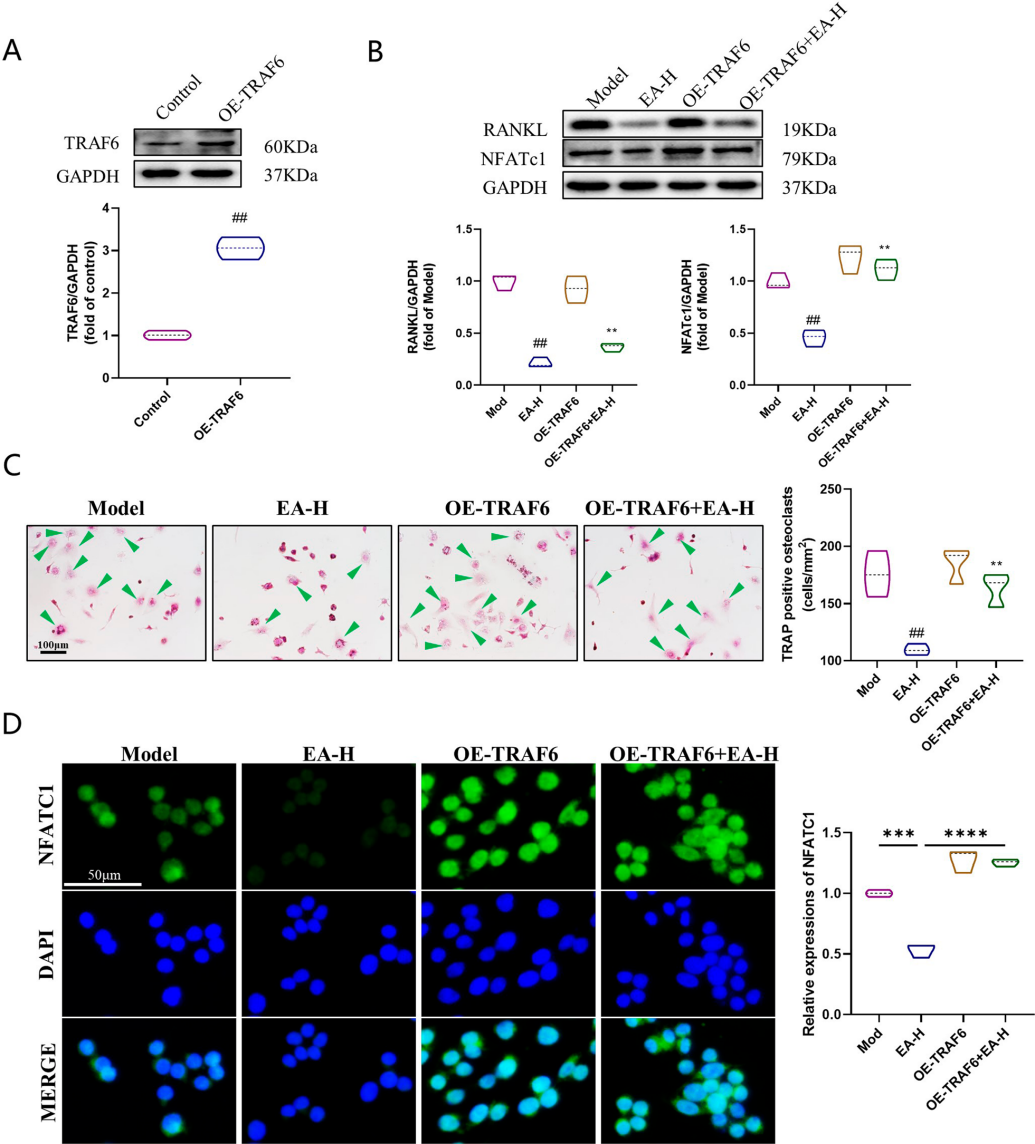
EA inhibits the proliferation and differentiation of OCs via TRAF6. **a** Western blot analysis of osteoclasts to measure TRAF6 expression. n = 3 for experimental replicates for each group. **b** WB evaluation of protein expression of NFATc1. n = 3 for experimental replicates for each group. **c** Osteoclast differentiation was detected by Trap staining in each group. n = 3 for experimental replicates for each group. **d** Representative images of immunofluorescence analysis of NFATc1. Scale bar = 50 μm. n = 3 for experimental replicates for each group. ^##^P < 0.01 and **P < 0.01 represent significant differences, ns indicates not significant

To determine whether EA inhibits TRAF6, we overexpressed TRAF6 using lentiviral vectors transfected into RANKL-induced osteoblasts. The effect of TRAF6 overexpression was determined by immunoblotting (Fig. 5a), and the expression of TRAF6 was significantly reduced (p < 0.01). As shown in Fig. 5b, NFATc expression was significantly downregulated in the EA group compared to the RANKL/M-SCF-treated group (p < 0.01), whereas in the TRAF6 high-expression group, the high dose of EA did not alter the expression of NFATc (p > 0.01). In other words, EA suppressed NFATc by inhibiting TRAF6, and these results were confirmed by immunofluorescence (Fig. 5c). Cellular experiments also showed that EA inhibited osteoclast formation via TRAF6 (Fig. 5d).

## Discussion

In this study, our data demonstrated that EA inhibited osteoclastogenesis in vitro via the TRAF6-dependent PI3K/Akt and NF-κB signalling pathways. Furthermore, EA reversed ovariectomy-induced osteoporosis in rats. In conclusion, these findings suggest that EA may be a novel drug for the treatment of osteoporosis.

With the advancement of osteoporosis research, therapeutic interventions have evolved from basic supplementation of bone minerals to strategies focused on stimulating bone formation. These interventions can impede osteoclast differentiation, diminish bone resorption, ameliorate bone microarchitecture, increase bone density, and mitigate fracture susceptibility. Alendronate is the first bisphosphonate drug approved by the U.S. Food and Drug Administration (FDA) for the treatment of postmenopausal osteoporosis. Alendronate has a strong affinity for hydroxyapatite in bone and exerts an antiresorptive effect by inhibiting osteoclast activity; however, this drug has side effects such as gastrointestinal reactions, so there is a need for a natural drug with fewer side effects.

Epimedium, a well-established herbal remedy with a history spanning centuries, has been traditionally utilized for fortifying bones, muscles, and tendons and for addressing conditions such as rheumatic diseases, cardiovascular diseases, and diabetes (Liu et al. 2021; Zhang et al. 2008). A recent study showed that epimedium medication effectively improves stromal cytokine culture in vivo, prompting bone marrow stromal cells to generate stem cell factors. This change, in turn, fosters cellular metabolism and protein synthesis, crucial for cellular proliferation and osteoblast differentiation, which are important processes (LeBoff et al. 2022). Notably, epimedium contains EA, one of its active components, in relatively high concentrations (Zhai et al. 2013) and has been found to show excellent efficacy in senile osteoporosis (Zhang et al. 2022). Consequently, our initial exploration focused on evaluating the potential of EA in osteoporosis treatment. Alendronate can inhibit the process of osteoblastogenesis, reduce and prevent osteoporosis, and has been used as a positive control in vivo.

Here, we used EA in an ovariectomy-induced osteoporosis model, which was evaluated by PCR, micro-CT and immunohistochemistry. An intact trabecular bone structure increases bone strength and reduces fracture occurrence (Sukhdeo et al. 2020). Micro-CT scans of the femur showed that EA significantly normalized bone microstructure and bone density in osteoporotic rats. To further illustrate or demonstrate the reason for the normalization in bone microstructure and BMD, we obtained TRAP-stained femur sections. The results showed that TRAP activity was inhibited after EA treatment. This finding suggested that EA improves bone microstructure and bone density by modulating the differentiation of osteoclasts. The above results indicate that EA can effectively increase bone strength and significantly normalize bone microstructure in osteoporotic rats and suggest that the inhibition of osteoclast differentiation reduces bone loss in vivo.

To further investigate the mechanism by which EA leads to increased bone mass, we performed in vitro experiments. Extensive osteoclastogenesis is widely recognized as a pivotal factor in the development of osteoporosis among postmenopausal women and elderly individuals (Fink et al. 2019; Jones et al. 2020). The primary pathological characteristics of osteoporosis include increased osteoclastogenesis and bone resorption (Black and Rosen 2016). Osteoclast differentiation is intricately linked with the upregulation of specific genes in response to RANKL signalling, and RANKL signalling inhibition is a proven therapeutic approach for the treatment of osteoclast-related diseases (Zhai et al. 2013). Consequently, comprehensively elucidating the underlying mechanisms governing osteoclast differentiation to gain insights into the aetiology of osteoporosis is urgently needed.

M-CSF and RANKL are two pivotal factors essential for osteoclastogenesis, affecting osteoclast differentiation, proliferation, prevention of apoptosis, lifespan extension, promotion of maturation, and the induction of osteoclast marker genes such as TRAP (Győri and Mócsai 2020). Therefore, in this study, in vivo model RAW264.7 cells were induced to form osteoclasts using medium containing M-CSF and RANKL. When RANKL binds to its receptor RANK, the complex interacts with TRAF6 and subsequently activates downstream pathways such as NF-κB and MAPK signalling. Previously, TRAF6-mediated NF-κB inhibition and PI3K/AKT stimulation were reported to prevent bone loss in ovariectomized mice (Liu et al. 2018; Ma et al. 2018). Hence, we posited the existence of an upstream regulatory molecule modulating the effects elicited by EA stimulation. In this study, we found that EA inhibited TRAF6, NFATc1, pAKT/AKT and pNF-κB/κB expression downstream of RANK, suppressed osteoclast differentiation, and effectively delayed oxidative stress-induced osteoporosis, consistent with previous studies.

Furthermore, when TRAF6 was overexpressed, the upregulation of its expression led to low expression of NFATc1, eliminating the inhibitory effect of EA on osteoclast differentiation. As a result, NFATc1 was identified as a central regulator of osteoclast differentiation (Xie et al. 2022). Mechanistically, we found that EA inhibits RANKL-induced activation of NF-κB signalling by blocking the phosphorylation of p65. Our findings emphasize that the EA-induced protective effect against bone loss relies on TRAF6.

There are several limitations to this study. First, we did not fully explore the potential mechanism by which EA is downregulated during RANKL-induced osteoclast formation. EA may inhibit osteoclasts through other pathways, and this issue will be a key focus of our future research. Second, the exploration of the potential effects of EA on osteoclasts was carried out with macrophages, and we did not assess osteoblasts, which require further study. Finally, this study did not investigate whether the inhibition of osteoclasts by EA after TRAF6 knockout was synergistically increased, which needs to be further studied. In the future, clinical trials are also needed to verify the ameliorative effect of EA on osteoporosis.

## Conclusions

Collectively, our results indicate that EA can impede osteoclastogenesis and RANKL-triggered signalling pathways in vitro and mitigate ovariectomy-induced bone loss in vivo. Moreover, EA inhibited osteoclast differentiation in vitro through the TRAF6-dependent PI3K/Akt and NF-κB signalling pathways and decelerated osteoporosis progression in the OVX rat model. These observations suggest that EA shows promise as a novel therapeutic agent for osteoporosis treatment.

## Data Availability

The datasets used and/or analysed during the current study are available from the corresponding author upon reasonable request.

## Abbreviations

EA: Epimedin A
TRAF6: TNF Receptor Associated Factor 6
PI3K: Phosphoinositide 3-Kinase
AKT: Protein Kinase B
NF-κB: Nuclear Factor kappa-light-chain-enhancer of activated B cells
RANKL: Receptor Activator of Nuclear Factor Kappa-B Ligand
M-CSF: Macrophage Colony-Stimulating Factor
TRAP: Tartrate-Resistant Acid Phosphatase
NFATc1: Nuclear Factor of Activated T-cells, Cytoplasmic 1
PCR: Polymerase Chain Reaction
ALN: Alendronate
BMD: Bone Mineral Density
BV/TV: Bone Volume/Tissue Volume Ratio
Tb.Th: Trabecular Thickness
Tb.N: Trabecular Number
Tb.Sp: Trabecular Separation
MAPK: Mitogen-Activated Protein Kinase
OC: Osteoclast
DAPI: 4',6-diamidino-2-phenylindole
CCK-8: Cell Counting Kit-8

## Declarations

### Studies in humans and animals

We expressly declare that we comply with these regulations, including the ARRIVE guidelines and the U.K. Animals (Scientific Procedures) Act, 1986 and associated guidelines, EU Directive 2010/63/EU for animal experiments, or the National Research Council’s Guide for the Care and Use of Laboratory Animals.
